# PCC0208057 as a small molecule inhibitor of TRPC6 in the treatment of prostate cancer

**DOI:** 10.3389/fphar.2024.1352373

**Published:** 2024-03-18

**Authors:** Yingjie Wei, Min Li, Yuemiao Hu, Jing Lu, Lin Wang, Qikun Yin, Xuechuan Hong, Jingwei Tian, Hongbo Wang

**Affiliations:** ^1^ School of Pharmacy, Key Laboratory of Molecular Pharmacology and Drug Evaluation (Yantai University), Ministry of Education, Collaborative Innovation Center of Advanced Drug Delivery System and Biotech Drugs in Universities of Shandong, Yantai University, Yantai, China; ^2^ Key Laboratory of Combinatorial Biosynthesis and Drug Discovery (MOE) and Hubei Province Engineering and Technology Research Center for Fluorinated Pharmaceuticals, Wuhan University School of Pharmaceutical Sciences, Wuhan, China

**Keywords:** prostate cancer, TRPC6 channel, TRPC6 small molecule inhibitor, cycle arrest, growth inhibition

## Abstract

Prostate cancer (PCa) is a common malignant tumor, whose morbidity and mortality keep the top three in the male-related tumors in developed countries. Abnormal ion channels, such as transient receptor potential canonical 6 (TRPC6), are reported to be involved in the carcinogenesis and progress of prostate cancer and have become potential drug targets against prostate cancer. Here, we report a novel small molecule inhibitor of TRPC6, designated as PCC0208057, which can suppress the proliferation and migration of prostate cancer cells *in vitro*, and inhibit the formation of Human umbilical vein endothelial cells cell lumen. PCC0208057 can effectively inhibit the growth of xenograft tumor *in vivo*. Molecular mechanism studies revealed that PCC0208057 could directly bind and inhibit the activity of TRPC6, which then induces the prostate cancer cells arrested in G_2_/M phase *via* enhancing the phosphorylation of Nuclear Factor of Activated T Cells (NFAT) and Cdc2. Taken together, our study describes for the first time that PCC0208057, a novel TRPC6 inhibitor, might be a promising lead compound for treatment of prostate cancer.

## 1 Introduction

Prostate Cancer (PCa) is one of the most common malignant tumors of the male genitourinary system, which has a long course and is prone to invasion and metastasis in the later stage (Jadvar, 2011; Center et al., 2012). According to the estimated cancer statistics of the United States in 2022, among 1.9 million cancer cases, the diagnosis rate of prostate cancer is about 27%, and the mortality rate is about 10% (Siegel et al., 2022). The early development of prostate cancer is controlled by androgen growth mechanism, and hormone therapy may be effective at this stage which can lead to tumor regression. When the prostate cancer metastasizes to bone and lymph nodes, the proliferation of prostate cancer cells at this time is controlled both by androgen-dependent and androgen-independent mechanisms, which results in uncontrollable expansion and invasion of the tumor (Prevarskaya et al., 2007; Schatten, 2018). However, there is currently no effective treatment against this proliferation. Thus, identification of new molecular targets of prostate cancer and development of effective treatment methods based on these targets are important to improve clinical prognosis of prostate cancer patients.

Enhanced cell proliferation, abnormal differentiation, and uncontrolled death lead to abnormal growth of tissue cells, which eventually transform into uncontrolled expansion and invasion. This transformation process is usually accompanied by changes in the expression of ion channels, resulting in abnormal expression of processes related to ion channels in cell processes (Gkika and Prevarskaya, 2011). More and more evidence has shown that the development of prostate cancer is related to the abnormal expression and functional changes of ion channels (Lastraioli et al., 2015; Farfariello et al., 2021).

Transient receptor potential canonical (TRPC) channels, which belong to the TRP family, are non-selective Ca^2+^ permeable cation channels expressed in various tissues (Zhang et al., 2018; Guo and Chen, 2019; Wang et al., 2020). TRPC6 has been reported to involve the regulation of cardiac hypertrophy, pulmonary vascular tone and permeability, blood pressure, renal fibrosis, and Alzheimer’s disease (Dietrich et al., 2005; Li et al., 2005; Wu et al., 2010; Paez Espinosa et al., 2012; Tauseef et al., 2012; Lin et al., 2019; Su et al., 2020). TRPC6 also plays a key role in the carcinogenesis, and is over-expressed in breast cancer, gastric cancer, glioblastoma (Li et al., 2005; El Boustany et al., 2008; Aydar et al., 2009; Cai et al., 2009; Ding et al., 2010). Studies have found that the expression of TRPC6 in prostate cancer cells is closely related to tumor stage and grade, suggesting that TRPC6 channels might be a novel anti-cancer drug target against prostate cancer (Yue et al., 2009).

Very few compounds have been reported as TRPC6 inhibitors (Urban et al., 2012; Urban et al., 2016; Bernichtein et al., 2017; Chen et al., 2017; Li et al., 2017; Zhang et al., 2018; Zhou et al., 2018; Diez-Bello et al., 2019; Lin et al., 2019). SKF-96365, a non-selective TRPC6 antagonist with the IC_50_ value of 4.9 μM has been reported to inhibit the Ca^2+^ elevation regulated by TRPC6 channels (Merritt et al., 1990; Inoue et al., 2001; Cai et al., 2009). The IC_50_ values of specific TRPC6 antagonists, SAR-7334, BI 749327, DS88790512, and SH045 were 7.9, 13, 11 and 5.8 nM, respectively (Maier et al., 2015; Hafner et al., 2018; Motoyama et al., 2018; Lin et al., 2019; Bai et al., 2020). Although these TRPC6 antagonists have reported nanomolar level activities *in vivo*, they have not been used in *vivo* studies, presumably due to their low potency and poor bioavailability (Lin et al., 2019).

Here we report a novel potent TRPC6 small-molecule antagonist, designated as PCC0208057. This compound can effectively inhibit the growth of prostate cancer cells growth both *in vitro* and *in vivo*, demonstrating the therapeutic potential of TRPC6 antagonists in treating prostate cancer.

## 2 Materials and methods

### 2.1 Materials

PCC0208057 (molecular formula: C_22_H_23_N_3_O_4_S; MW: 425.51) was synthesized and obtained as a light-yellow powder. Purity of the compound used in the present study was greater than 98% as confirmed by HPLC. The synthesis process of PCC0208057 is provided in Supplementary Material. SKF-96365 (molecular formula: C_22_H_26_N_2_O_3_; MW: 402.91) was synthesized and obtained as a light-yellow powder. Purity of the compound used in the present study was greater than 98% as confirmed by HPLC. Docetaxel was purchased from Qilu Pharmaceutical Co., LTD. (Shandong, China). PCC0208057 and SKF-96365 were dissolved in DMSO and stored at −20°C for less than 1 mouth before use in *vitro* experiments. For *in vivo* experiments, PCC0208057 was dissolved in cremophor EL and absolute ethanol (v: v = 1 : 1) by sonication, then diluted in saline (v: v = 1 : 3) immediately before use. Docetaxel was diluted in saline (v: v = 1 : 9) before administration. Anti-TRPC6 antibody (ab62461) was obtained from Abcam. NFATc3 (F-1) (sc-8405) and p-NFATc3 (C-3) (sc-365786) were obtained from Santa Cruz Biotechnology. Cdc2 (POH1) Mouse mAb (#9116) and Phospho-cdc2 (Thr14) Antibody (#2543) were obtained from Cell Signaling Technology.

### 2.2 Cell culture and animals

Human prostate cancer cell lines (LNCaP and PC3) were purchased from the cell of Chinese Academy of Sciences (Shanghai, China) and cultured in RPMI-1640 media supplemented with 10% fetal bovine serum (FBS, Excell Bio, Shanghai, China), penicillin (100 U/mL) and streptomycin (100 μg/mL). Human umbilical vein endothelial cells (HUVEC) were purchased from ATCC and cultured in RPMI-1640 media supplemented with 10% FBS, penicillin (100 U/mL) and streptomycin (100 μg/mL). All cells were grown in 37°C with 5% CO_2_ and harvested during the exponential growth phase. Male BALB/c Nude mice were purchased from Vital River Laboratory Animals Technology (Beijing, China). The animals were housed in a light- and temperature-controlled room (21°C–25°C; relative humidity 55%–60%) and maintained on a standard food and water. All animal studies comply with the ARRIVE guideline, and all of the experimental protocols were approved by the Committee of the Ethics of Animal Experiments of Yantai University (Yantai, China).

### 2.3 Molecular docking

The 3D complex of hTRPC6 bound to AM-1473 (PDB ID: 6UZA) was used for molecular docking. The proteins were optimized by removing all water molecules in the crystal strcture, adding hydrogens, endowing with CHARMm force field in Discovery Studio 2018. The benzothiazole amides were pretreated with energy minimization. The active site was defined as the sphere around the cognate ligand with a diameter of 10 Å. The remaining ligand-protein docking parameters were used as default settings.

### 2.4 Cell proliferation assay

The proliferation of cell was estimated by determination of cell growth curves and clone formation assay (Zhang et al., 2022). For the cell growth curve assay, cells were plated into 6-well plates (8,000/well) and cultured overnight. Cells were then treated with the tested compounds for 24 h. Growth curve was obtained by counting cells over six consecutive days. For the colony formation assay, cells were plated into 6-well plates (1,000/well) and incubated overnight. Cells were then treated with the tested compounds for 14 days. After 14 days of seeding, the colonies were stained with 0.5% crystal violet and clones were counted.

### 2.5 Fluorescent calcium ions and electrophysiological experiments

Cells were loaded with Fluo4-AM (Invitrogen) to monitor intracellular Ca^2+^ changes, and changes in membrane potential were monitored using a FlexStation microplate reader (molecular device) (Wei et al., 2021). The fluorescence changes were also read in the microculture plate reader with the addition of PCC0208057 and HDM. The trace represents the reading of three repeated measurements at the beginning of the experiment (F_0_), which was normalized to the mean fluorescence change (ΔF) of fluorescence (repeated three times). At the same time, the concentration response curve of PCC0208057 for inhibiting TRPC6 was determined by Ca^2+^ assay (Fluo-4). The change in fluorescence intensity was expressed as the ratio of the change in fluorescence to the change in fluorescence before application of the stimulus or test compound.

Electrophysiological measurements were performed by changing the concentration of compound PCC0208057 in TRPC6 cells. The voltage command was regulated by the PatchMaster program (version 2.60, HEKA) and the current was recorded at 5 kHz. The voltage was held from 0 mV to 100 mV in 20 m and from −100 mV to 100 mV in 20 m–100 m. By means of a gravity-driven multichannel system, the bath solution was continuously perfused into the cell at the desired channel at a distance of 50 μm from the cell, and the current-voltage (I-V) relationship was obtained by a voltage ramp. The concentration-response curves of PCC0208057, which inhibited TRPC6, were determined by electrophysiological recordings.

### 2.6 Flow cytometry assay

The cells were seeded into six well plates (2×10^5^, 1.5×10^5^, 8×10^4^ cells/well) and cultured overnight (Ma et al., 2018), followed by treatment with various concentrations of the tested compounds for 24, 48 h. Then cells were harvested and fixed with cold 80% alcohol overnight at 4°C, were washed in PBS, and treated with Cell Cycle Detection Kit for 37°C for 30 min. Cell cycle phase distributions were analyzed by a flow cytometer (BD Biosciences, SanJose, CA, USA).

### 2.7 Western blotting assay

Cells were treated with the tested compounds for 16 h and then lysed in RIPA cell lysis buffer (Li et al., 2019). After lysis, they were centrifuged at 15,000 rpm using the Eppendorf centrifuge for 15 min at 4°C. Equal amounts of protein (20 μg) were subjected to SDS-PAGE and transferred onto PVDF membranes. The membranes were blocked with 5% fat-free dried milk in TBST buffer for 2 h and incubated overnight with primary antibodies. After being incubated with the corresponding secondary antibodies, the proteins were visualized using an enhanced chemiluminescence system, following the protocol for the BeyoECL Plus.

### 2.8 Cell migration assay

Cells were seeded in six well plates with serum-free media at a concentration of 5×10^5^/well and cultured overnight (Li et al., 2020). The cell monolayers were scratched using sterile 200 μL pipette tips. Subsequently, the cells were treated with various concentrations of the test compounds, which were diluted in serum-free medium. After both 0 and 24 h, three randomly selected fields were photographed and the migration distance was analyzed using PS software.

### 2.9 Transwell assays

Transwell migration assays were performed using six well Transwell chambers (3464, Corning, NY, USA) with 8 μm permeable pores, following the manufacturer’s instructions (Ding et al., 2018). Cell were seeded in the chamber with serum-free medium and various concentrations of test compounds, while the space beneath the chamber was filled with complete medium. The cells were allowed to migrate for 24 h. Afterward, the cells on the surface of the membrane were carefully washed with PBS and any remaining cells were removed with a cotton swab. The cells adhering to the membrane were then fixed and stained with crystal violet. The stained cells were visualized and counted from three randomly selected fields using a fluorescent inverted microscope. The migration distance was analyzed using PS software to analyze.

### 2.10 Lumen formation assay

The 24-well plate was coated with 100 μL of Matrigel matrix (356,234, Corning) and incubated for 30 min at 37°C. HUVEC (8×10^4^ cells/well) were seeded onto the Matrigel bed and treated with various concentrations of the test compounds in the presence of VEGF (30 ng/mL) for 24 h. Tube formations were observed using an inverted microscope and the tubular structures were counted manually (Ding et al., 2018).

### 2.11 *In vivo* anti-cancer efficacy studies

The LNCaP cells were harvested, washed, and suspended in PBS. Then 2×10^6^ cells were injected subcutaneously into the right flank regions of the mice (Hu et al., 2023). When the tumors reached an average volume of 100–300 mm^3^, the mice were randomized into five groups (n = 6/group): a) Control; b) 10 mg/kg Docetaxel; c) 50 mg/kg PCC0208057; d) 100 mg/kg PCC0208057. Both the control group and PCC0208057 groups were injected intraperitoneally with respective solutions every day, while Docetaxel was injected intraperitoneally every 3 days. Tumor diameters and body weight were measured every 3 days during the treatment, and the relative tumor volume was calculated using the formula: L × W)^2^/2. At the end of the treatment, the mice were euthanized, the xenograft tumors were removed and weighed, and the rate of tumor inhibition for each treatment was then calculated based on the tumor weight.

### 2.12 Statistics

All the experiments were performed at least 3 times, and all the values were expressed as mean ± SD. Statistical analysis was carried out using *t*-test for comparison between two groups. One-way ANOVA followed by Dunnett’s test was used to analyze the data involving three or more groups. *p < 0.05* was considered statistically significant.

## 3 Results

### 3.1 Synthesis of PCC0208057

**FIGURE 1:**
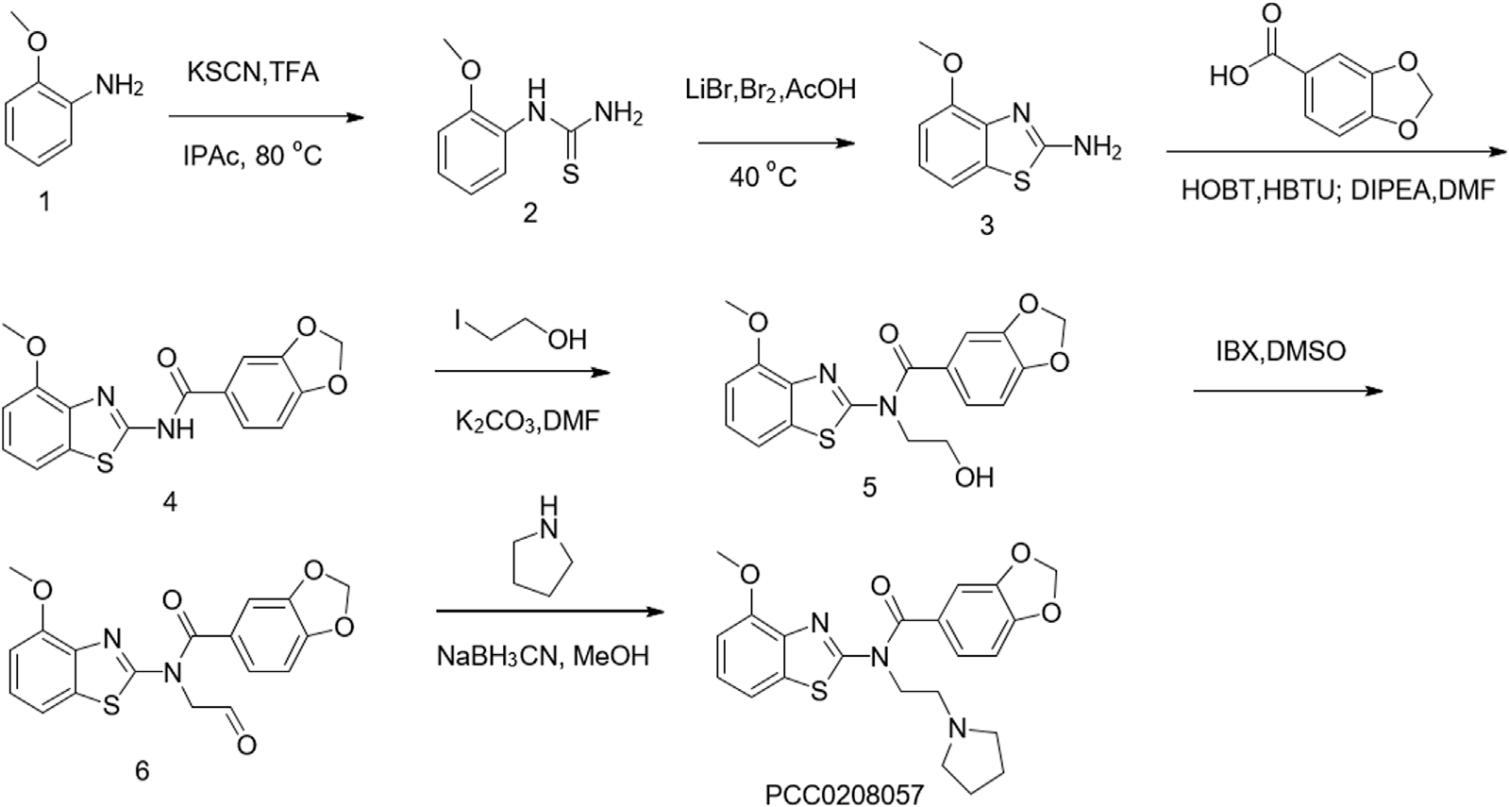
Chemical synthesis of PCC0208057.

The synthesis of PCC0208057 is shown in Figure 1.

### 3.2 PCC0208057 inhibits agonist-mediated TRPC6 activity

**FIGURE 2:**
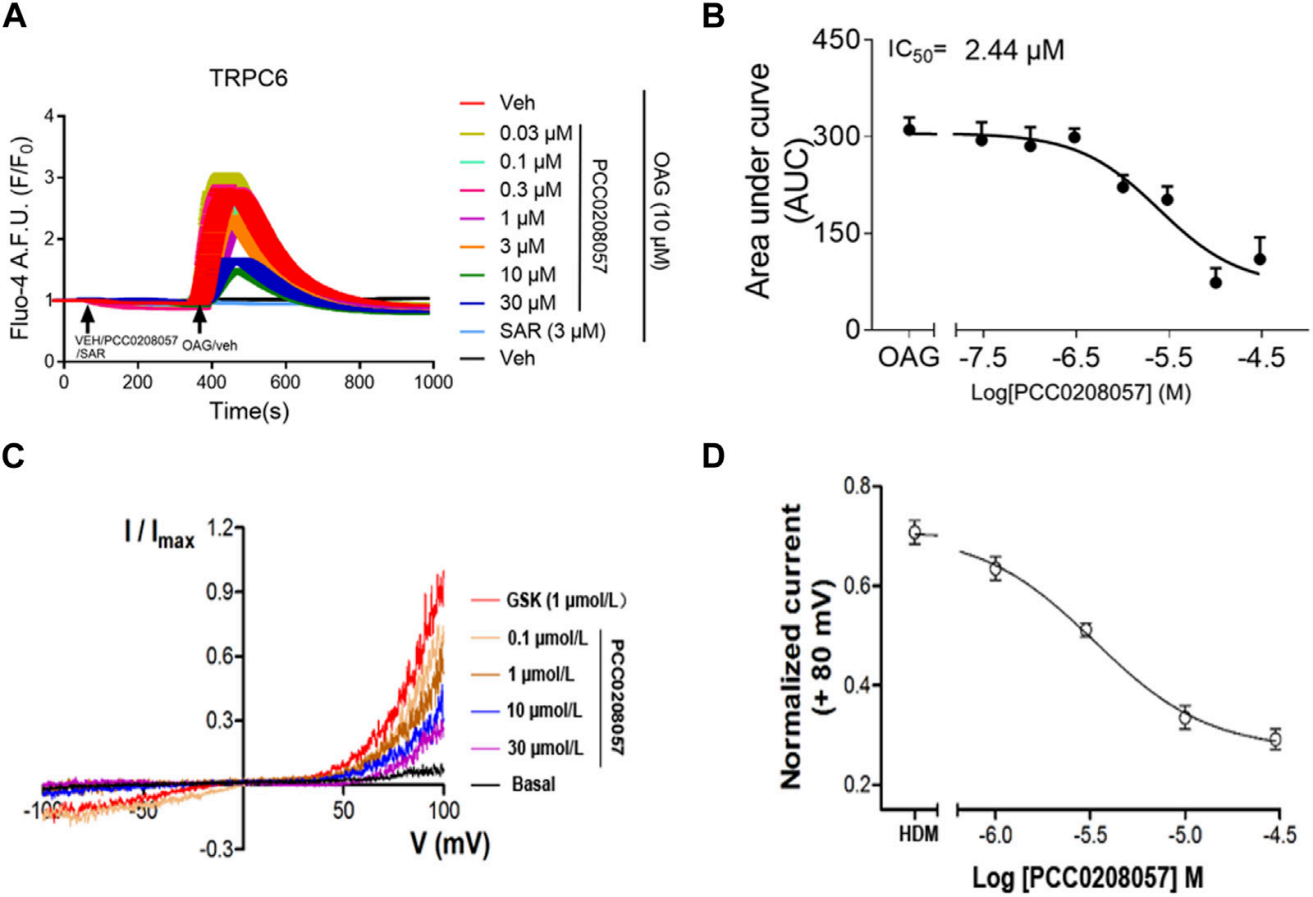
PCC0208057inhibitedagonist-mediated TRPC6 activity. **(A)** PCC0208057 inhibited the Ca^2+^ response in TRPC6-HEK293 cells induced by SAR7334. **(B)** The concentration-response curves of compound PCC0208057 in inhibiting TRPC6 was determined by Ca^2+^ assay (Fluo-4). The solid lines indicated the fitting of the Hill equation, which obtained the IC_50_ values. **(C)** By changing the concentration of compound PCC0208057 in the TRPC6 cell, the current-voltage (I-V) relationships were acquired through the voltage ramps. GSK1702934A (1 μmol/L) activated current and PCC0208057 inhibited current. **(D)** The concentration-response curves for compound PCC0208057 determined by electrophysiology recording to inhibit TRPC6. The solid lines were represented by the Hill equation, which produced the IC_50_ value.

The effect of PCC0208057 on TRPC6 channel function was detected by fluorescent calcium ion and electrophysiological experiments. Results as shown in Figure 2 A and B, PCC0208057 inhibited the increase of [Ca^2+^]_i_ caused by TRPC6 agonist SAR7334 (3 μmol/L) in TRPC6 expressing cells, and the inhibitory effect was proportional to the concentration. These results indicated that PCC0208057 inhibited TRPC6-mediated Ca^2+^ concentration increase in a concentration-dependent manner, and the IC_50_ values of compound PCC0208057 on TRPC6 inhibition was 2.44 μmol/L. Similarly, electrophysiological experiments showed that PCC0208057 inhibited TRPC6 current changes induced by the non-specific TRPC6 agonist GSK 1702934A (1 μmol/L), and the higher the concentration, the more obvious the inhibitory effect (Figure 2 C and D).

### 3.3 PCC0208057 binds directly to TRPC6

**FIGURE 3:**
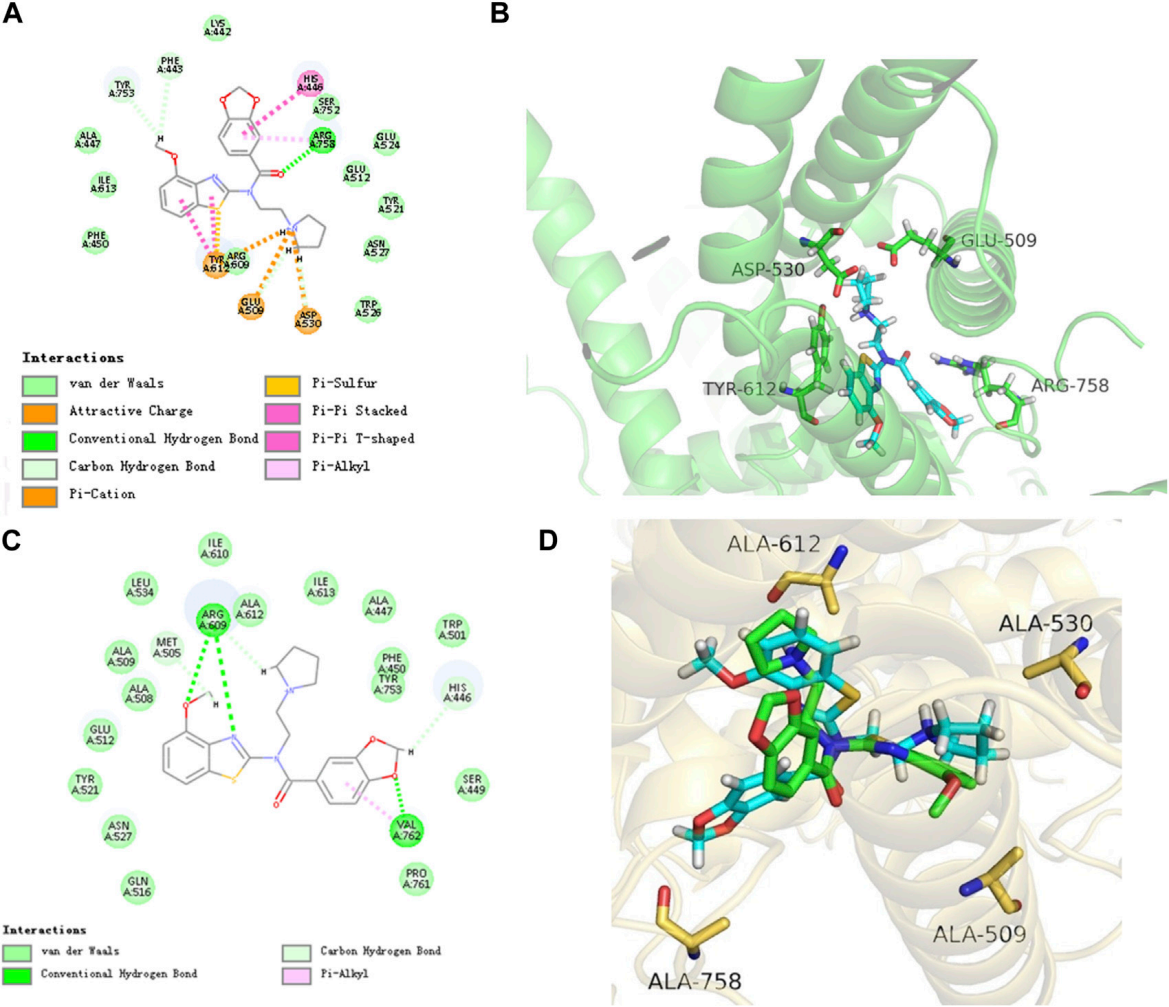
Binding mode of PCC0208057, TRPC6 channel and mutant TRPC6 channel. **(A)** 2D docking structure of PCC0208057. **(B)** Binding pattern of PCC0208057 to TRPC6. **(C)** 2D docking structure of PCC0208057. **(D)** Binding pattern of PCC0208057 to mutant and wild type TRPC6.

The results of molecular docking experiments showed that the N atom of the pyrrole ring of PCC0208057 formed ionic bonds with Glu509 and Asp530, the carbonyl group formed hydrogen bonds with Arg758, and the benzo [d] thiazole nucleus of PCC0208057 had π-cation interaction with Tyr612. Thus, the stable binding pattern in the *in vitro* assay was demonstrated (Figure 3 A and B). Then we have mutated the four residues of TRPC6 (E509A, D530A, R758A, Y612A) using SYBYL-X2.1.1, and performed the molecular docking of PCC0208057 on these TRPC6 mutants. The docking results (Figure 3 C and D) indicated that the conformation of PCC0208057 was flipped in the target, and only formed weak hydrophobic interactions with A509 and A612.

### 3.4 PCC0208057 inhibits the proliferation of prostate cancer cells

**FIGURE 4:**
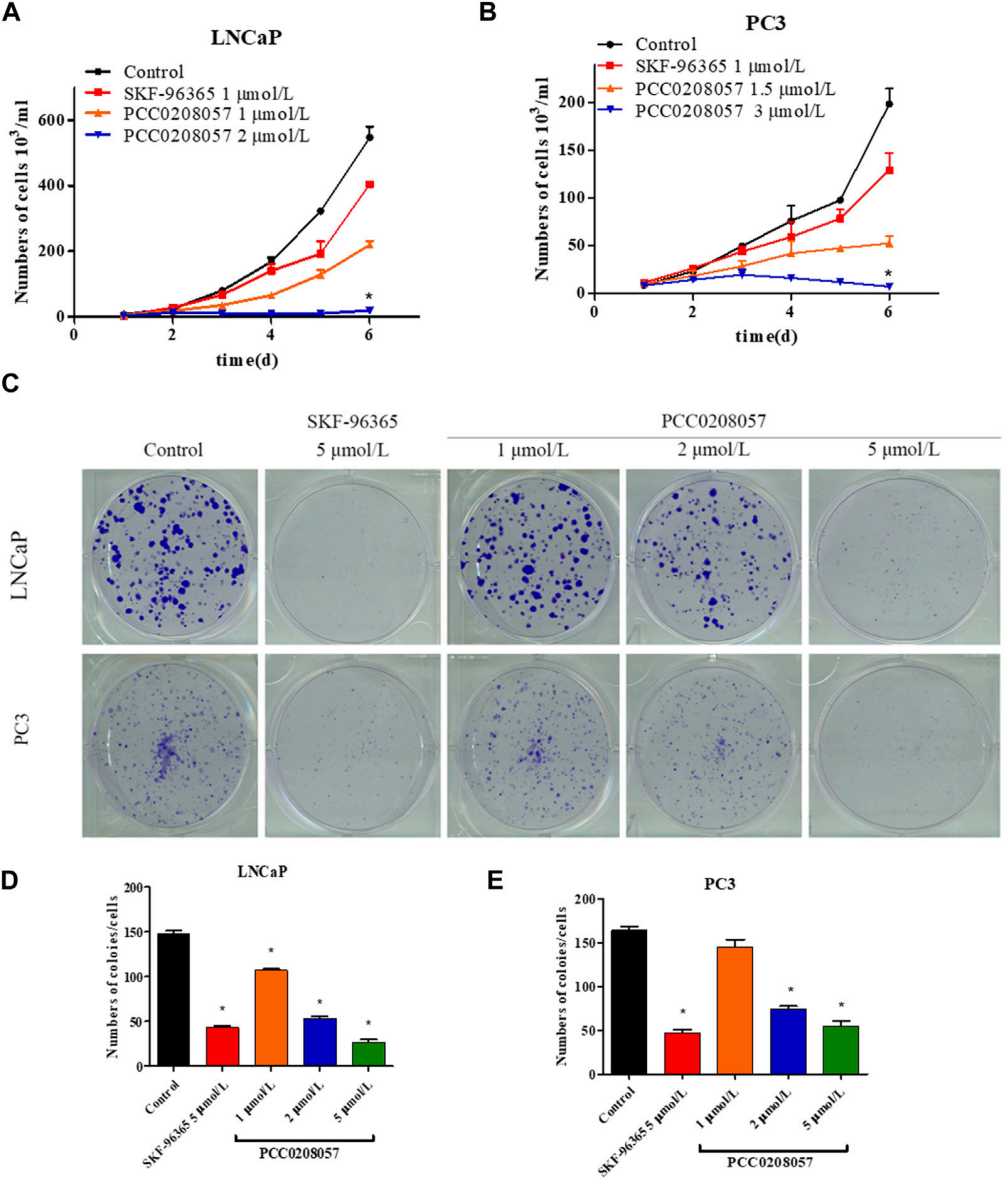
The effect of PCC0208057 on the cell proliferation of prostate cancer cells. Data were normalized to that in the absence of drug treatment from the same experiment and expressed as means ± SD of three experiments. *p < 0.05, compared with control group.

Consistent with these findings, both cell growth curves and clone formation assay, showed that PCC0208057 decreased the growth and colony formation in LNCaP and PC3 cell lines in a dose-dependent manner (Figures 4A–E). The *in vitro* inhibitory activity of PCC0208057 was better than or equivalent to that of SKF-96365 when assayed under the same conditions.

### 3.5 PCC0208057 induced prostate cancer cell cycle arrest at G_2_/M phase

**FIGURE 5:**
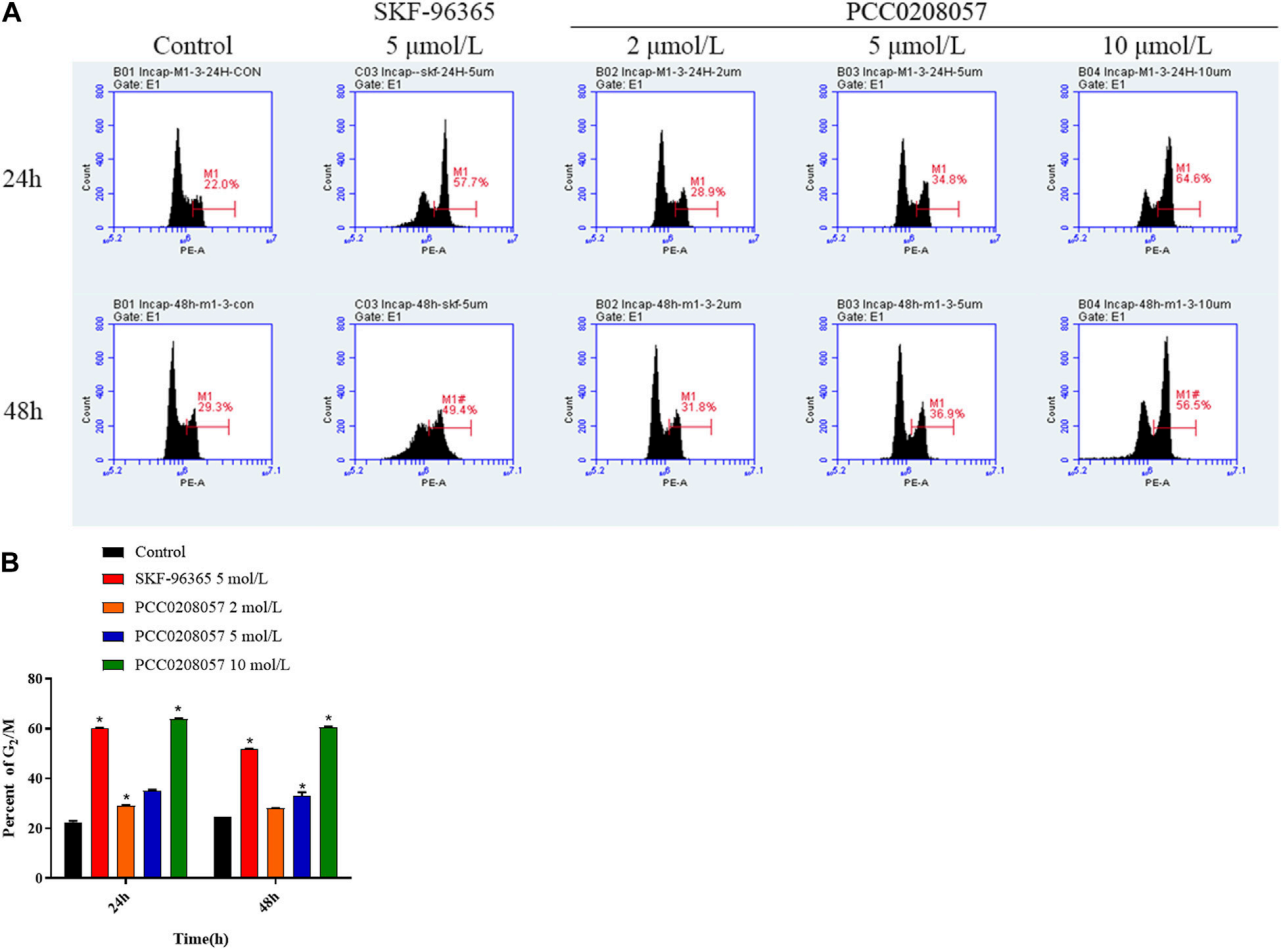
The effect of PCC0208057 on the cell distribution of LNCaP cells. Data were normalized to that in the absence of drug treatment from the same experiment and ex-pressed as means ± SD of three experiments. *p < 0.05, compared with control group.

Flow cytometry was used to detect the effect of PCC0208057 on LNCaP cells cycle. Compared with the control group, the ratio of cells in G_2_/M phase was significantly increased after 24 and 48 h treatment with PCC0208057, and the higher the drug concentration, the more the proportion of G_2_/M phase cells. The results showed that PCC0208057 could block the cycle of prostate cancer cells (Figure 5 A and B).

### 3.6 PCC0208057 increased the phosphorylation of NFAT and Cdc2

**FIGURE 6:**
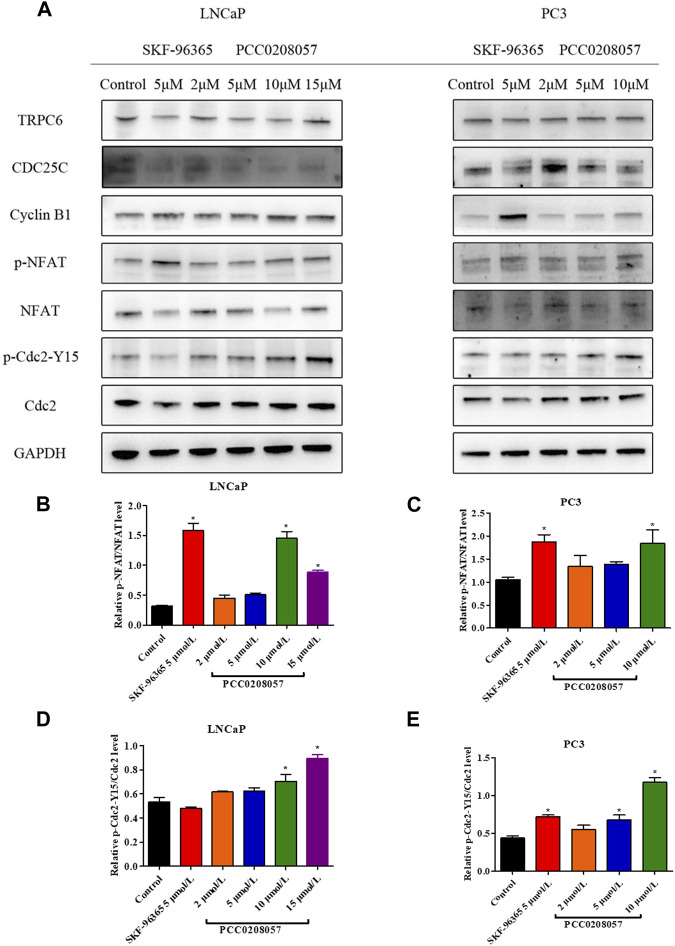
Effects of PCC0208057 on NFAT and Cdc2 in prostate cancer cells. **(A)** PCC0208057 affected the expression of related proteins in LNCaP and PC3 cells; **(B)** Gray value analysis of p-NFAT/NFAT protein expression in LNCaP cells; **(C)** Gray value analysis of p-NFAT/NFAT protein expression in PC3 cells; **(D)**Gray value analysis of p-Cdc2-Y15/Cdc2 protein expression in LNCaP cells; **(E)** Gray value analysis of p-Cdc2-Y15/Cdc2 protein expression in PC3 cells. Data were normalized to that in the absence of drug treatment from the same experiment and are means ± SD of three experiments. *p < 0.05, compared with control group.

Western blot assay was then used to detect the effect of PCC0208057 on NFAT signal pathway in LNCaP cells and PC3 cells. Compared with the control group, PCC0208057 could increase the phosphorylation level of NFAT and Cdc2 at Tyr15 (Figures 6A–E). No obvious changes on the expression of TRPC6 were observed after incubation for 16 h.

### 3.7 PCC0208057 inhibited the migration of prostate cancer cells

**FIGURE 7:**
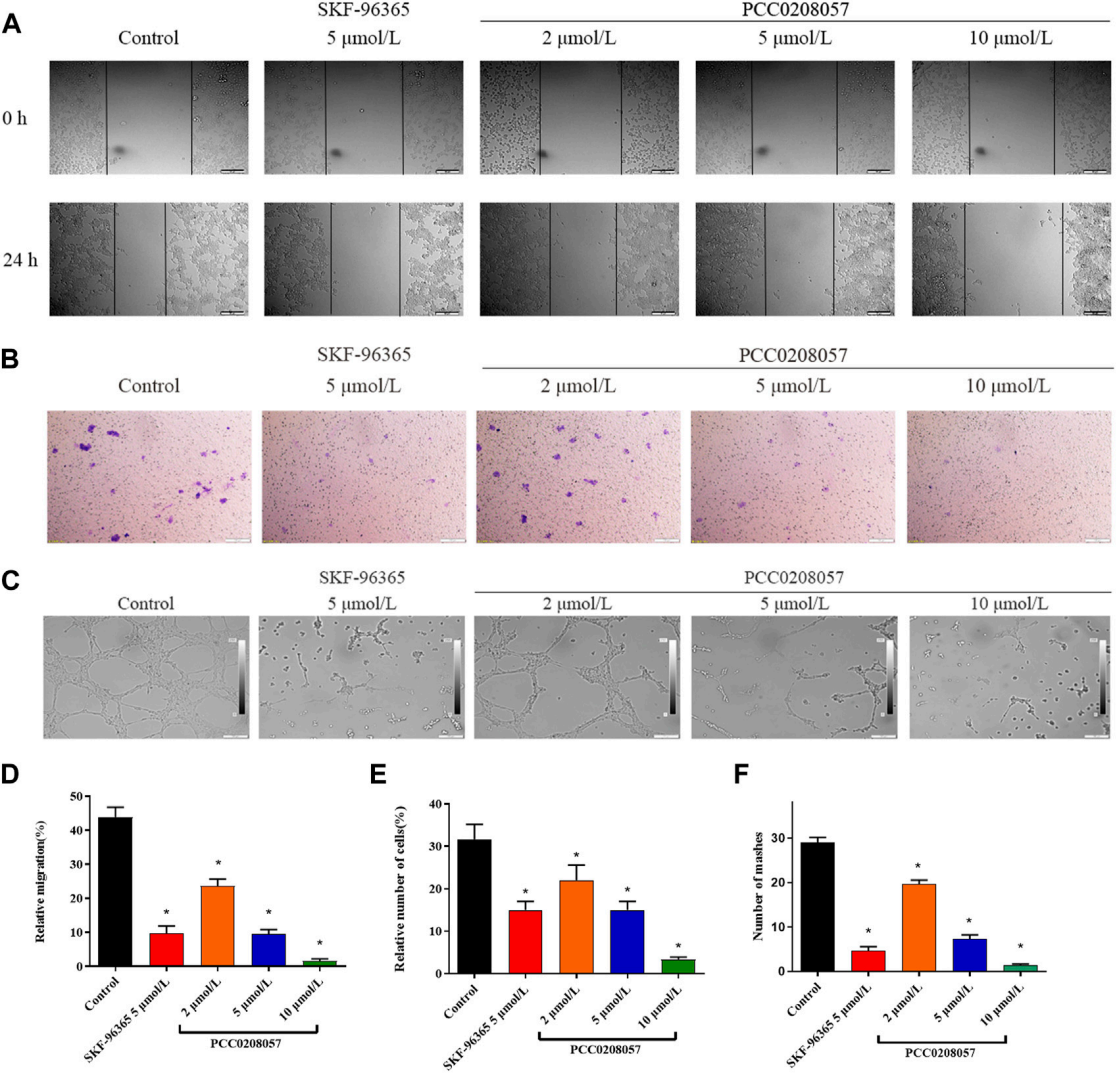
The effect of PCC0208057 on the migration, invasion and lumen formation. **(A)** LNCaP cells were treated with PCC0208057 at the indicated concentrations. Scratches were made and images were taken at 0 and 24 h and the migration distances were summarized. **(B)** Formation of LNCaP cells through the Transwell chamber. **(C)** The lumen formation of HUVEC cells. **(D)** Statistics of relative migration distances of LNCaP cells. **(E)** Relative cell number statistics of LNCaP cells with compartments. **(F)** Lumen formation statistics of HUVEC cells. Data were normalized to that in the absence of drug treatment from the same experiment and expressed as means ± SD of three experiments. *p < 0.05, compared with control group.

Scratch test was used to demonstrate the effect of PCC0208057 on the migration ability of LNCaP cells. Results were shown in Figure 7 A and D. As compared to the control group, the inward migration degree of cells in scratches was reduced after treatment with PCC0208057, and the higher the concentration, the lower the migration degree. These results indicated that PCC0208057 could inhibit the inward migration of prostate cancer cells.

### 3.8 PCC0208057 inhibited the invasion and metastasis of prostate cancer cells

Transwell assay was used to detect the effect of PCC0208057 on the invasion and metastasis ability of LNCaP cells. As shown in Figure 7 B and E, when compared to the control group, the number of cells transferred to the outside of the small room in the drug treatment group decreased, and the higher the concentration, the less the number of cells. These results indicated that PCC0208057 could inhibit the invasion and metastasis of prostate cancer cells.

### 3.9 PCC0208057 inhibited the lumen formation of HUVEC cells

Lumen formation assay is a dynamic *in vitro* assay that provides a key step in angiogenesis, including proliferation of vascular endothelial cells and formation of tubular vessel structures. To further explore the effect of PCC0208057 on VEGF-induced angiogenesis, the effect of PCC0208057 on HUVEC lumen formation was examined. As shown in Figure 7 C and F, the lumen formation of HUVEC cells was inhibited after drug treatment for 24 h, and the higher the drug concentration, the more obvious the lumen formation was inhibited. And the results showed that the compound did not cause cytotoxicity (Supplementary Figure S2).

### 3.10 PCC0208057 had good antitumor efficacy in nude mice

**FIGURE 8:**
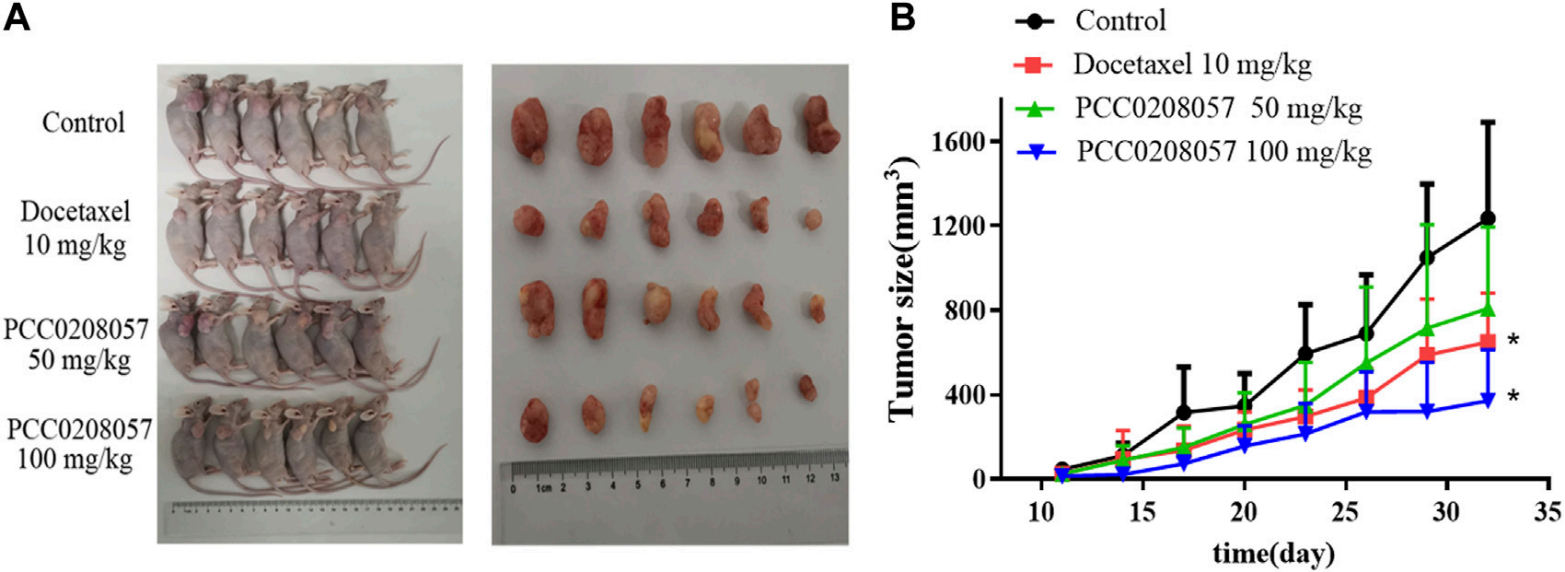
Antitumor effects of PCC0208057 in LNCaP tumor model. **(A)** The representative photographs of tumors after last treatment in each group were taken; **(B)** Tumor size. Data were normalized to that in the absence of drug treatment from the same experiment and expressed as means ± SD (n = 6). *p < 0.05, compared with control group.

To explore the antitumor activity of PCC0208057 *in vivo*, LNCaP xenograft tumor model was established in BALB/c nude mice. As shown in Figure 8 A, 20 days after administration, PCC0208057 at doses of 50 mg/kg and 100 mg/kg inhibited the growth of LNCaP xenografts in a dose-dependent manner (*p < 0.05*, compared with control group). Low dose (50 mg/kg) of PCC0208057 had no apparent effect on the body weight of mice (Table 1, *p* > *0.05*, compared with control group) while high-dose (100 mg/kg) of PCC0208057 significantly decreased the body weight in mice (Table 1, *p < 0.05*, compared with control group), suggesting that compound PCC0208057 may have certain toxic effect at high dose (100 mg/kg). Importantly, PCC0208057 has clear and significant antitumor effect at the lower non-toxic dose (50 mg/kg). The tumor suppression rates of 50 mg/kg and 100 mg/kg PCC0208057 were 41.88% and 74.36%, respectively (Table 1, *p < 0.05*, compared with control group).

**TABLE 1:** Antitumor effects of PCC0208057 in LNCaP tumor models.

Group (mg/kg)	Number (initial/End)	Body weight (g)		Tumor weight (g)	
		Initial	End	g	IR (%)
Control	6/6	22.68 ± 0.75	25.6 ± 1.36	1.17 ± 0.23	
Docetaxel	6/6	23.82 ± 1.24	24.99 ± 1.95	0.39 ± 0.16^a^	66.67^a^
10					
PCC0208057	6/6	23.9 ± 1.09	25.23 ± 2.14	0.68 ± 0.35^a^	41.88^a^
50					
PCC0208057	6/6	23.37 ± 1.46	20.68 ± 3.36^a^	0.3 ± 0.17^a^	74.36^a^
100					

^a^ *p < 0.05*, compared with control group.

## 4 Discussion

Prostate cancer is a male genitourinary tumor with the high incidence and mortality among male cancers. Although it can be alleviated by surgical or chemical castration, it eventually turns into an uncontrollable androgen insensitive prostate cancer. There is currently no effective treatment for this irreversible development. Here, we report a new structural compound PCC0208057, that could be used as a potential lead compound in the treatment of prostate cancer.

**FIGURE 9:**
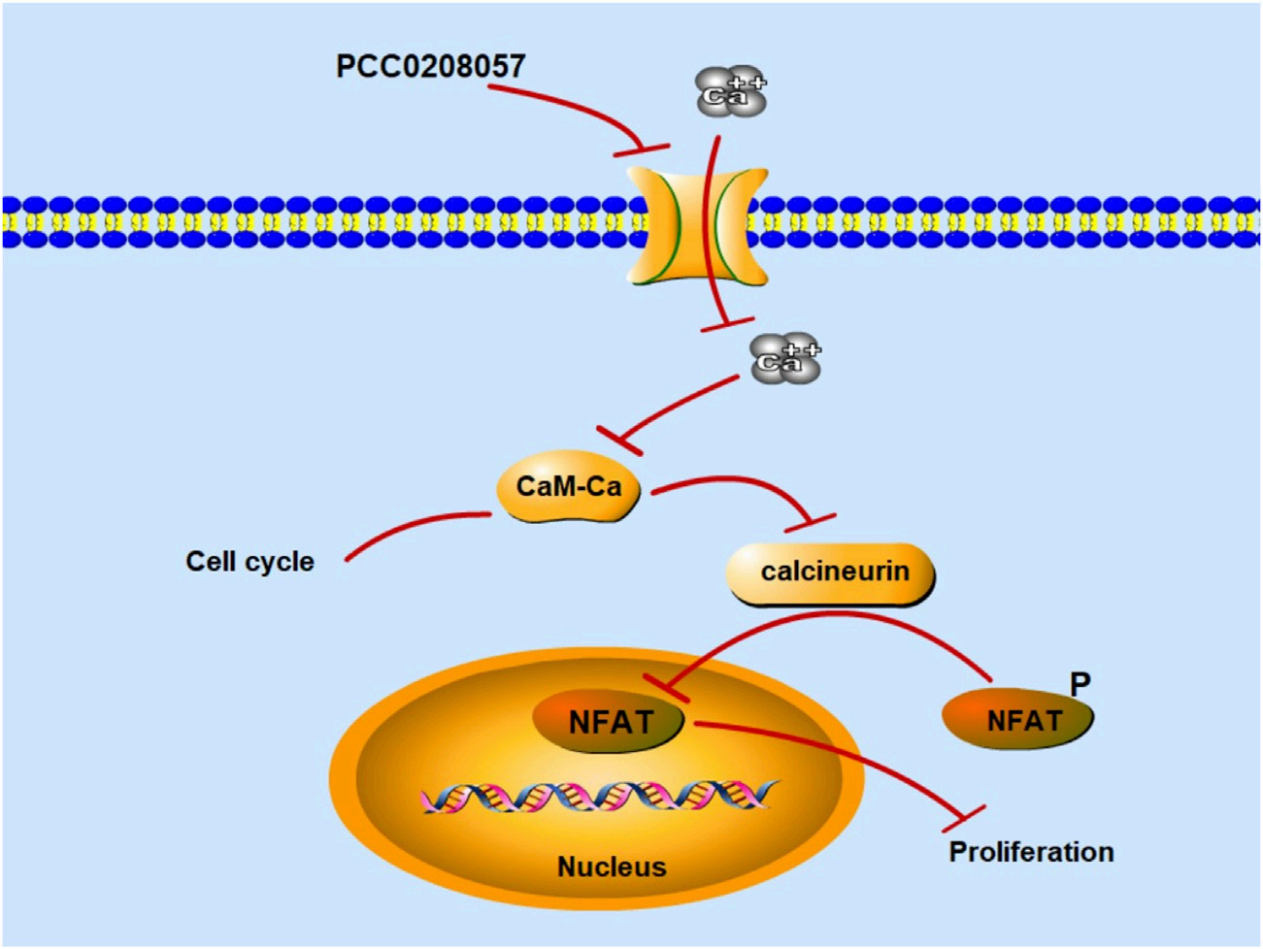
Mechanism diagram of PCC0208057 in the prostate cancer. PCC0208057 inhibits Ca^2+^ entry through TRPC6 channel, inactivates calcineurin in the CaM-Ca^2+^ complex pathway, inhibits dephosphorylation of p-NFAT in cytoplasm, and fails to combine with downstream genes for transcription in the nucleus, thus inhibiting the proliferation of cancer cells.

Inhibition of TRPC6 activity by pharmacological agents can suppress the proliferation of cancer cells (Wang et al., 2010). TRPC6 channel reportedly could regulate the concentration of Ca^2+^ through the Ca^2+^ signaling pathway mediated by PLC signal and then affect cell proliferation through a series of reactions (Figure 9) (Thebault et al., 2006). Increased intracellular Ca^2+^ levels induce the activation of calcineurin in the CaM-Ca^2+^ complex pathway, which dephosphorylates many proteins, including activated T cytokines (NFAT), and dephosphorylates NFAT phosphorylated in the cytoplasm to the nucleus, where NFAT is transcribed by binding to downstream genes (Muller and Rao, 2010). Molecular docking experiments showed that PCC0208057 directly combined with TRPC6. At the same time, after TRPC6 gene was silenced by siRNA gene silencing, the expression of TRPC6 protein was decreased, and the inhibitory effect of PCC0208057 on the proliferation of prostate cancer cells was weakened compared with the non-silenced cells (Supplementary Figure S1). The results of Western blot experiments showed that PCC0208057 increased phosphorylated NFAT expression and decreased non-phosphorylated NFAT expression by reducing TRPC6 expression in LNCaP and PC3 cells. The results showed that PCC0208057 indeed inhibited Ca^2+^ influx through the TRPC6 channel, and thus inhibited the NFAT signaling pathway that relied on Ca^2+^ entry to play transcriptional and proliferative roles. Studies have shown that Ca^2+^, as the second messenger of cells, plays an important role in cell cycle regulation. Cdc2 plays an important role in phase transitions in phase G_2_/M. Blocking TRPC6 can inhibit Ca^2+^ influx, and inhibiting or downregulating TRPC6 can cause a series of responses, including increased phosphorylation at the Tyr15 site of Cdc2. The results of Western blot experiments showed that when the expression of TRPC6 was inhibited with PCC0208057, the phosphorylation level of Tyr15 site of Cdc2 increased while the level of total Cdc2 protein remained largely unchanged. Meanwhile the increase of phosphorylation level at the Tyr15 site of Cdc2 also indicate that PCC0208057 affected the entry of Ca^2+^ regulated by the TRPC6 channel through the CaM-Ca^2+^ complex pathway and at the same time affected the expression of periodic protein in the G_2_/M phase, which in turn led to phase transition in the G_2_/M phase. The results of Western blot show that PCC0208057 can affect Cdc2 expression, so we speculate that prostate cancer cells may develop G_2_/M phase block after treatment with PCC0208057. Therefore, flow cytometry was used to study the effect of PCC0208057 on the prostate cancer cell cycle. Indeed, PCC0208057 could block prostate cancer cells in the G_2_/M stage, indicating that PCC0208057 can lead to cycle arrest of prostate cancer cells.

Cell migration is a process that involves multiple physiological and pathological processes, including wound healing, cancer cell metastasis, *etc.* Actin cytoskeleton and cell adhesion structures play an important role during cell migration. The different amplitudes of Ca^2+^ signals in cells effectively control the changes in different parts of migrating cells. The involvement of TRP channels in cell migration has been reported and TRPC6 channels mainly affect cell migration by regulating Ca^2+^ signaling (Fiorio Pla and Gkika, 2013). In this study, the inhibitory effect of PCC0208057 on the migration and invasion of prostate cancer cells was confirmed using scratch experiment and Transwell experiment. The experimental results showed that PCC0208057 could inhibit the scratch repair ability and invasion and metastasis ability of prostate cancer cells, suggesting that PCC0208057 can inhibit prostate tumor cell migration, invasion, and metastasis.

Angiogenesis is a crucial process for the development and growth of cancer. The formation of new blood vessels not only provides sufficient oxygen and nutrients for tumor growth, but also promotes tumor spread and metastasis (Negri et al., 2019). Studies have shown that TRPC6 channels are involved in VEGF-induced luminal formation of HUVEC cells. VEGF can increase intracellular Ca^2+^ concentration by inducing extracellular Ca^2+^ entry and intracellular Ca^2+^ release, and promote angiogenesis by stimulating the TRPC6 channel. At the same time, the activation of the TRPC6 channel initiates a cascade reaction that migrates endothelial cells (Ge et al., 2009). To explore the anti-angiogenesis potential of PCC0208057, lumen formation assay was adopted and the effect of PCC0208057 on lumen formation of HUVEC cells was tested using concentrations with minimal effect on HUVEC cell proliferation. The experimental results showed that PCC0208057 could inhibit the formation of HUVEC cell lumen, indicating that PCC0208057 can inhibit angiogenesis through TRPC6 channel which may contribute to its antitumor potential.

In a previous study, 1s, a compound that shares similar chemical structure with PCC0208057, did not have anti-tumor activity *in vivo* (Wei et al., 2021). To investigate the antitumor potential of PCC0208057, we established LNCaP xenograft tumor model in BALB/c nude mice. The results showed that PCC0208057 at doses of 50 mg/kg and 100 mg/kg could inhibit the growth of LNCaP xenografts in a dose-dependent manner, with tumor inhibition rates of 41.88% and 74.36%, respectively. Although the high dose of PCC0208057 had certain toxicity related to body weight loss in the animals, it clearly showed that PCC0208057 can effectively inhibit the growth of tumor in nude mice *in vivo*.

In conclusion, PCC0208057, a small molecule inhibitor of TRPC6, directly binds to TRPC6 and inhibits its activity. This likely leads to cell cycle arrest through the CaM-Ca^2+^ complex pathway, the NFAT signaling pathway and the modulation of the expression of Cdc2. Ultimately, it demonstrated clear antitumor efficacy against prostate cancer both *in vitro* and *in vivo*. These results suggest that TRPC6 channel can be a new drug target for prostate cancer treatment, and PCC0208057 can be a potential candidate compound.

## Data Availability

The original contributions presented in the study are included in the article/Supplementary Materials, further inquiries can be directed to the corresponding authors.
