# Supplementary material for: PCC0208057 as a small molecule inhibitor of TRPC6 in the treatment of prostate cancer
Source: Front Pharmacol. 2024 Mar 18;15:1352373. doi: 10.3389/fphar.2024.1352373 (PMC10986179; doi:10.3389/fphar.2024.1352373)
Supplement: Supplementary file 1 [file DataSheet1.DOCX]

^[[1]](#endnote-1)^Supplementary Material

# Supplementary Data

# 1.1 Chemical synthesis of compound PCC0208057.

Take 100 mL round-bottled flask, add o-methoxyaniline (i.e., compound 1, 4.00g, 32.48 mmol), dissolve with 50 mL sec-butyl acetate, add KSCN (4.73 g, 48.72 mmol), stir for 10 min, then add trifluoroacetic acid (9.26 g, 9.26 g) drop by drop under ice bath. 81.20 mmol), after adding trifluoroacetic acid, stir at room temperature for 1 h, and heat up to 80 ℃ and stir overnight. TLC checks if the reaction is complete. After the reaction was stopped, the reaction liquid was stirred in an ice bath for 2 h, a large amount of white solid was precipitated, and the white solid was extracted by pumping and filtration, then washed with 20 mL for 3 times, and the white solid was dried in a vacuum drying oven at 40 ℃, and 4.00g white powder (intermediate 2, yield 67%) was obtained.

The intermediate 2 (1.00g, 5.49 mmol) was added to 100 mL round-bottomed flask, 20 mL glacial acetic acid was added to stir and dissolve, lithium bromide (0.72g, 8.24 mmol) was added at room temperature, the reaction system was moved to 0 ℃ ice bath, and liquid bromine (0.28mL, 5.49 mmol) was slowly added. Remove to room temperature and stir for a period of time. Heat to 40 ℃ and react overnight. After the reaction, the reaction liquid was moved to room temperature and stirred for 2 h, the white solid was extracted and filtered, washed with 20 mL acetic acid three times, and the white solid was dried in a vacuum drying oven at 40 ℃ overnight, and 0.80 g of white powder (intermediate 3, 80% yield) was obtained.

Intermediate 3(0.50 g, 2.77 mmol), piperic acid (0.46 g, 2.77 mmol), HOBT(0.56 g, 4.16 mmol) and HBTU(1.58 g, 4.16 mmol) were added into a 50 mL round-bottled flask. Add 30 mL of resteamed DMF to dissolve, stir at room temperature for 30 min, slowly add DIPEA (2.18 mL, 12.48 mmol), and stir at room temperature for one day. After TLC detection, the impurities were found to be very light in color. The reaction solution was diluted with 120 mL ethyl acetate, extracted with 50 mL water for 5 times to wash off the excess DMF, the organic phase was washed with 40 mL saturated salt water, dried with anhydrous MgSO4, and then separated by column chromatography after spin drying. PE: EA=4:1 to PE: EA=2:1 to PE: EA=1:1, the white solid 0.43 g (intermediate 4, yield 44.7%).

The intermediate 4 (0.10 g, 0.31 mmol) was added to 25 mL round-bottomed flask, dissolved with 10 mL DMF, added K2CO3 (0.13 g, 0.92 mmol), slowly dropped iodized ethanol (0.16 g, 0.92 mmol), and the reaction system was moved to 50 ℃ and stirred overnight. The reaction of TLC was complete. The reaction solution was diluted with 40 mL ethyl acetate, and the excess DMF was extracted with 30 mL water for 5 times. The organic phase was washed with 15 mL saturated salt water, and dried with anhydrous MgSO4 for 1 h, and then separated by column chromatography after spin drying. EA=1:1, the white solid is 0.07g (intermediate 5, yield 61.9%).

The intermediates 5 (0.15 g, 0.40 mmol) and IBX (0.28 g, 0.81 mmol) were added to a 25 mL round-bottomed flask, dissolved with 5 mL DMSO and 5 mL toluene, and stirred at room temperature overnight. The reaction of TLC was complete. The reaction solution was diluted with 60 mL ethyl acetate, washed with 15 mL saturated Na2S2O3 for 3 times to remove excess IBX, extracted with 20 mL water for 3 times to remove excess DMF, washed with saturated salt solution for 5 mL, dried with anhydrous MgSO4 for 1 h, and then separated by column chromatography after spin drying. PE: DCM=1:1 to PE: DCM=1:2, the light red powder solid 0.12 g (intermediate 6, 80% yield).

The intermediate 6(0.11g, 0.22mmol) was added into a 10mL round-bottled flask, 4 mL DCM/MeOH mixture (v:v=1:1) was added, tetrahydropyrrole (0.11mg, 1.08mmol) was added, and the mixture was stirred at room temperature for 30 min. NaBH3CN (0.056g) was added. 0.66 mmol) at room temperature for 24 h. The reaction of TLC was complete, MeOH was removed by spin evaporation, dissolved with DCM 40 mL, washed with water (5 mL×3) to remove excess dimethylamine hydrochloride and NaBH3CN, the organic phase was washed with 15 mL saturated salt and dried with anhydrous Na2SO4 for 1 h, filtered, and the filtrate was spun dry through the column, PE: EA=2:1 (1% triethylamine) to PE: EA=1:1 (1% triethylamine), resulting in a light yellow solid of 0.06 g (i.e. PCC0208057, yield 47.62%).

# Supplementary Figures and Tables

## By silencing TRPC6 gene, PCC0208057 reduced the inhibitory effect on the proliferation of prostate cancer cells

SiRNA gene silencing technique was used to silence TRPC6 gene, and the protein expression after silencing TRPC6 gene was detected by Western Blot assay. The results (Figure S1 A and B) showed that after the silencing of TRPC6 gene, the expression of TRPC6 protein in both LNCaP and PC3 cells decreased. MTT assay was used to detect the effect of drugs on the proliferation of silenced cells. The results (Figure S1 C and D) showed that after silencing TRPC6 gene, the inhibitory effect of PCC0208057 on cell proliferation was weakened.


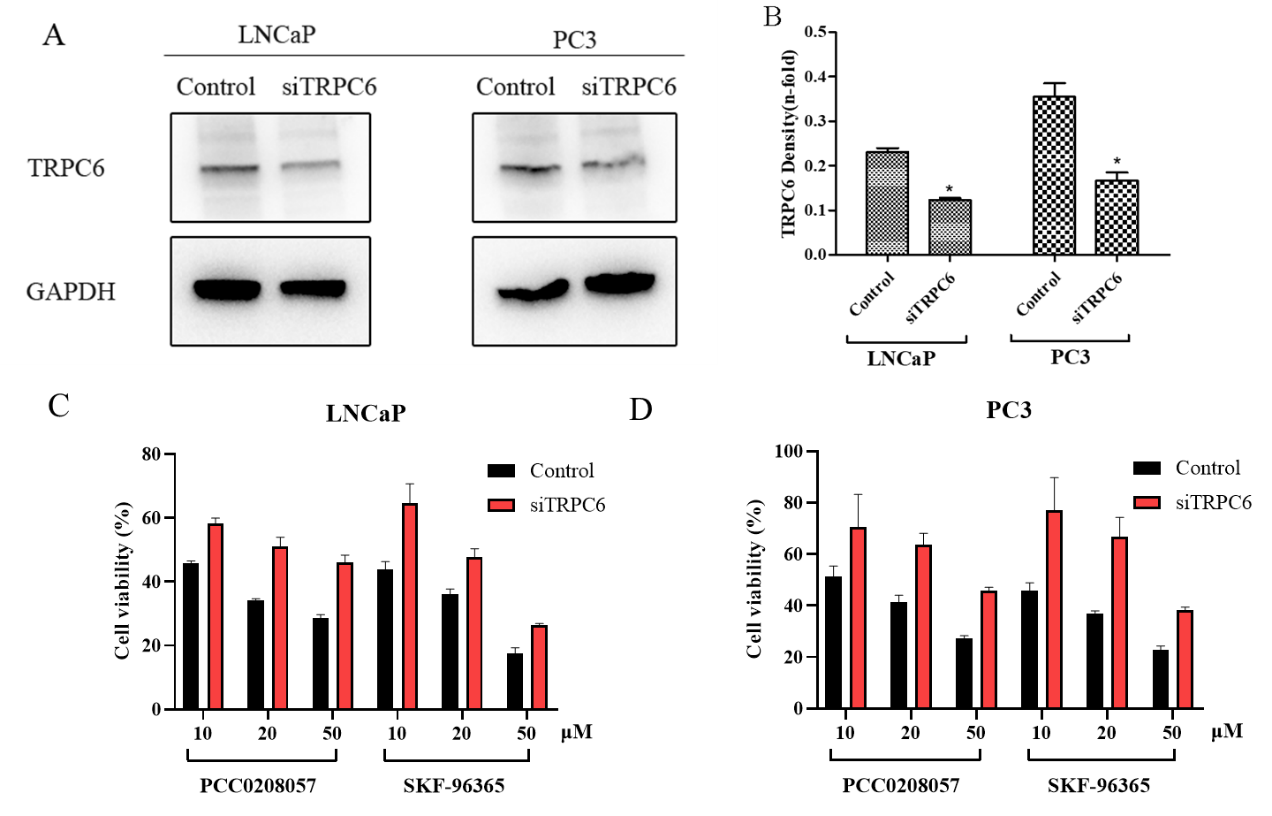


**Supplementary Figure S1.** Effect of PCC0208057 on TRPC6 protein expression and cell proliferation in prostate cancer cells after silencing TRPC6 by siRNA. **(A)** TRPC6 protein expression in LNCaP and PC3 cells after silencing TRPC6 by siRNA; **(B)** Gray value analysis of TRPC6 protein expression in LNCaP and PC3 cells after silencing TRPC6 by siRNA. **(C)** and **(D)** The effects of PCC0208057 and SKF-96365 on the proliferation of LNCaP and PC3 cells after silenced. All data are expressed as mean ± SD (n=3). All data are expressed as mean ± SD (n=3). **P＜0.05*, compared with Control group.

**PCC0208057 had no effect on the proliferation of HUVEC cells**

MTT assay was used to detect whether compound PCC0208057 inhibited lumen formation of HUVEC cells through cytotoxicity. The experimental results showed (Figure S2 A and B) that the compound had no significant inhibitory effect on cell proliferation, indicating that the compound did not inhibit lumen formation by inhibiting cell proliferation.


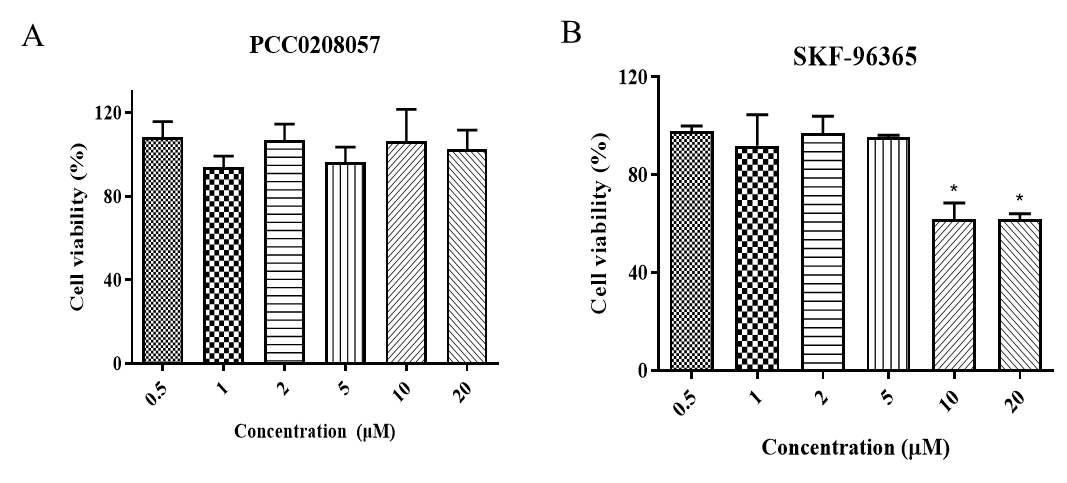


**Supplementary Figure S2.** The Effect of PCC0208057 on the proliferation of HUVEC cells. **(A)** The effect of PCC0208057 on the proliferation of HUVEC cells for 24 h; **(B)** The effect of SKF-96365 on HUVEC cell proliferation after 24 h treatment. All data are expressed as mean ± SD (n=3). **P＜0.05*, compared with 0.5 μM group.

1. [↑](#endnote-ref-1)
